# Efficacy and Safety of Neoadjuvant Targeted Therapy vs. Neoadjuvant Chemotherapy for Stage IIIA EGFR-Mutant Non-small Cell Lung Cancer: A Systematic Review and Meta-Analysis

**DOI:** 10.3389/fsurg.2021.715318

**Published:** 2021-08-19

**Authors:** Dong Chen, Zixian Jin, Jian Zhang, Congcong Xu, Kanghao Zhu, Yuhang Ruan, Bo Zhang, Baofu Chen, Jianfei Shen

**Affiliations:** ^1^Department of Thoracic Surgery, Taizhou Hospital of Zhejiang Province Affiliated to Wenzhou Medical University, Linhai, China; ^2^Department of Thoracic Surgery, Taizhou Hospital, Zhejiang University, Linhai, China

**Keywords:** non-small cell lung cancer, neoadjuvant therapy, chemotherapy, molecular targeted therapy, epidermal growth factor receptor, meta-analysis

## Abstract

**Purpose:** The role of targeted therapy in the neoadjuvant field of stage IIIA epidermal growth factor receptor (EGFR) mutation-positive non-small cell lung cancer (NSCLC) is still controversial. We sought to evaluate the efficacy and safety of neoadjuvant targeted therapy (NTT) with neoadjuvant chemotherapy (NCT) used as a benchmark comparator.

**Methods:** A systematic search was conducted in four databases (Pubmed, Cochrane Library, Embase, CNKI) for eligible studies on NTT published before October 2020. The primary endpoints were overall survival (OS), progression-free survival (PFS), objective response rate (ORR), and grade 3/4 adverse events (AEs). Statistical analysis and bias assessment were performed by RevMan 5.3.

**Results:** A total of 319 patients, including 3 randomized controlled trials and 2 non-randomized controlled trials, were included in the meta-analysis. Perform the second subgroup analysis after excluding 2 non-randomized controlled trials. The meta-analysis reveals that, for EGFR mutation-positive stage IIIA NSCLC patients, compared with NCT, NTT can significantly increase ORR (relative risk [RR]:1.70, 95% confidence interval [CI]:1.35–2.15; subgroup-RR:1.56, 95% CI 1.23–2.0) and significantly reduce grade 3/4 AEs (RR:0.5, 95% CI 0.34–0.75; subgroup-RR: 0.53, 95% CI 0.26–1.08). The OS of the NTT arm is slightly higher, but the difference is not significant (hazards ratio [HR]: 0.74, 95% CI: 0.43–1.27; subgroup-HR: 0.64 95% CI 0.40–1.03). No difference in PFS was found (HR: 0.81, 95% CI 0.27–2.44).

**Conclusion:** In neoadjuvant setting, targeted therapy has a definitive effect on patients with EGFR mutation-positive stage IIIA NSCLC and is even better than chemotherapy in terms of toxicity and tumor response rate.

**Systematic Review Registration:** PROSPERO, identifier CRD42021221136.

## Introduction

Lung cancer is the cancer with the highest rates of incidence and mortality in the world. According to the Global Cancer Statistics 2018, there are ~2.09 million new lung cancer cases and 1.76 million lung cancer deaths worldwide each year, accounting for 11.6 and 18.4% of global cancers, respectively (1). Non-small cell lung cancer (NSCLC) accounts for 80% of lung cancers, and epidermal growth factor receptor (EGFR) mutations are common in patients with NSCLC in Asian populations (the EGFR mutation rate of NSCLC patients in the white populations is 20%, and for Asian populations up to 50%) (2). As the disease progresses, the patient's survival prognosis drops sharply. According to statistics, the 5-year survival rates of patients with stage I, IIA, IIIA, and IV lung cancer are 90, 60, 36, and 10%, respectively (3). Stage III patients have a high risk of recurrence after surgery, and their standard treatment strategies have been controversial (4).

According to the NCCN guidelines (Version 1.2020), patients whose clinical assessment is potentially resectable stage IIIA NSCLC should be given induction chemotherapy first, and those who have not progressed during the treatment period undergo further surgery (5). After surgery, the patient will be classified into R0, R1, and R2 according to the surgical margin and receive treatments of Chemotherapy, Chemoradiation, and Concurrent chemoradiation, respectively. But with the advent of targeted therapy, the efficacy of EGFR tyrosine kinase inhibitors (TKI) in EGFR mutation-positive advanced NSCLC has been verified in a number of large-scale clinical trials. Several studies represented by CTONG-0802 suggest that EGFR-TKI conferred a significant PFS benefit in patients with EGFR mutation-positive advanced NSCLC and was associated with more favorable tolerability (6–8). According to the CTONG1104 trial report, in the adjuvant treatment phase of stage II to IIIa EGFR mutation-positive NSCLC, the disease-free survival (DFS) of the gefitinib group was significantly improved compared with the chemotherapy group, and gefitinib group's overall survival (OS) performance was gratifying (9). The phase 3 randomized ADAURA trial included completely resected stage IB to IIIA EGFR mutation-positive NSCLC patients, and showed that patients who received osimertinib had significantly longer DFS than those who received placebo (10). In the latest NCCN guidelines, the first-line treatment of EGFR mutation-positive advanced NSCLC patients has been replaced from chemotherapy to EGFR-TKI therapy. So can neoadjuvant targeted therapy (NTT) produce the same effect or even better?

Encouraged by the remarkable anti-tumor activity and acceptable toxicity of EGFR-TKI in the adjuvant treatment of lung cancer, some prospective studies have begun to explore the effectiveness and safety of EGFR-TKI in neoadjuvant therapy. Unfortunately, the sample size of the current study is limited and the conclusions are not completely consistent. Therefore, through this systematic review and meta-analysis, we will compare the advantages and disadvantages of NTT and neoadjuvant chemotherapy (NCT) in terms of the OS, progression-free survival (PFS), operation rate, grade 3/4 adverse events (AEs), and objective response rate (ORR). It is hoped that this can provide reference evidence for standard treatment in areas with a high incidence of EGFR mutations.

## Materials and Methods

### Search Strategy and Selection Criteria

According to the guidelines for systematic reviews and meta-analyses (PRISMA) (11), literature searches were conducted via Pubmed, Cochrane Library, Embase and CNKI databases up to October 2020. The search terms (Include synonyms) included: “lung neoplasm,” “non-small cell lung cancer or NSCLC,” “epidermal growth factor receptor or EGFR,” “neoadjuvant therapy,” “molecular targeted therapy,” “chemotherapy,” and specific drug names (See Supplementary Materials for search strategies). There were no language restrictions. The retrieved citations were initially and independently screened by the two authors (DC and JZ) through the title and abstract. All discrepancies were resolved by CX. We also checked the corresponding citations from the references to make up for deficiencies in the search. If the same clinical trial had been published in different journals or in different years, we selected the one with the most complete data.

The following are the inclusion criteria: (1) prospective clinical controlled trials about EGFR mutation-positive stage IIIA NSCLC patients; (2) compare NTT with NCT; (3) provide clinical data on the OS, PFS, ORR, AEs (12). The following are the exclusion criteria: (1) the trial includes patients outside of stage IIIa; (2) The patient received adiotherapy before surgery; (3) <20 patients were enrolled.

### Data Extraction

The primary endpoints were the OS, PFS, and ORR, as shown by RECIST version 1.1 (13). Secondary end points were the operation rate, progress rate, and grade 3/4 AEs. The following information was extracted from eligible studies by two authors (DC and ZJ), although some articles contained only partial data: first author, year of publication, number of incidents, total sample size, patient's tumor characteristics, treatment strategy, and hazard ratios (HRs) and 95% confidence intervals (CIs) of the OS and PFS. The ORR includes the complete response (CR) rate and the partial response (PR) rate. It was best to obtain data directly from the literature. If that could not be accomplished, we used Parmar's method to estimate data according to the chart included in the article (14).

### Assessment of Risk of Bias

We did not examine a funnel plot, because of the small number of studies. We used the RevMan version 5.3 (Nordic Cochrane Centre, Cochrane Collaboration, Copenhagen, Denmark) to evaluate the quality of eligible study according to the Cochrane Handbook for Systematic Reviews of Interventions. The risk of bias was assessed based on the following criteria: Random sequence generatio, Allocation concealment, Blinding of participants and personnel, Blinding of outcome assessment, In-complete outcome data, Other bias.

### Statistical Analysis

The meta-analysis of relative risk (RR) for the ORR and the meta-analysis of the HR for the OS and PFS were analyzed using Review Manager (RevMan) version 5.3. The two-sided Cl was set at 95%. *P* > 0.05 is considered not statistically significant. An RR of >1 meant that the event was more likely to occur in the experimental group (NTT); an HR of <1 meant fewer deaths and better prognosis in the experimental group (NTT), and vice versa. We evaluated the heterogeneity of the included literature according to the I-square (*I*^2^), and selected a suitable effects model. Because non-randomized controlled trials (non-RCTs) were included in the initial analysis, in order to reduce the interference of low-quality data with the results, the complete meta-analysis was performed twice. The first analysis included all of the literature, and the second considered only the RCT's data.

## Results

### Selection of Trials

**Figure 1:**
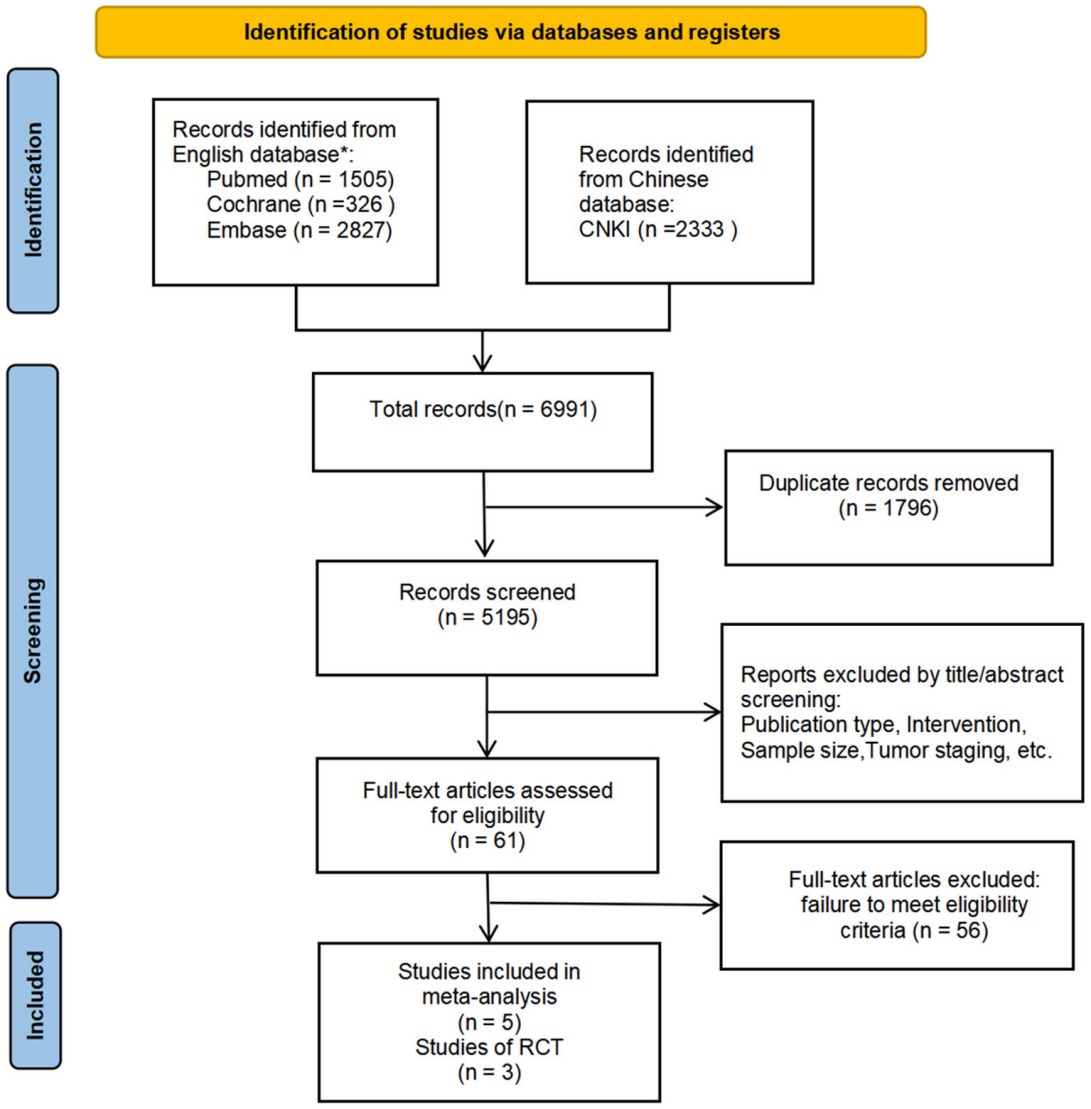
PRISMA Flow Diagram. We also checked the corresponding citations from the references to make up for deficiencies in the search. According to whether non-RCTs are included or not, two analyses are carried out separately to interpret the data more comprehensively. ^*^Relevant search terms are provided in Supplementary Materials.

Figure 1 illustrates the study retrieval process. A total of five articles, and 319 patients were included in the analysis (264 in the subgroup analysis) (Table 1). Three of the trials were qualified RCTs (16–18). All of the subjects were patients with EGFR mutation-positive stage IIIA NSCLC. The subjects were randomly assigned to the NTT group or NCT group. The other two non-RCTs were excluded to form a new subgroup for the second data analysis (15, 19). These two trials assign treatment strategies based on EGFR mutation status.

**Table 1:** Characteristics of included studies.

**Study**	**Study type**	**Cancer TNM**	**Participants**	**Population**	**HR[95%CI]**		**OR**		**Progression**		**Surgery**		**AEs**	
					**OS**	**PFS**	**NTT**	**NCT**	**NTT**	**NCT**	**NTT**	**NCT**	**NTT**	**NCT**
Zhong et al. (15)	Non-RCT	NSCLC IIIA-N2	A:Erlotinib(*N* = 12) B:GC-chemo(*N* = 12)	Total	1.79 [0.73–4.4]	2.26 [0.91–5.61]	7/12	3/12	3/12	2/12	6/12	7/12	2/12	3/12
Chen et al. (16)	RCT	NSCLC IIIA	A:Erlotinib(*N* = 43) B:PP-chemo(*N* = 43)	Mut EGFR	0.5 [0.24–1.07]	N.A.	29/43	19/43	2/43	5/43	39/43	36/43	9/43	14/43
Zhong et al. (17)	RCT	NSCLC IIIA-N2	A:Erlotinib(*N* = 37) B:GC-chemo(*N* = 35)	Mut EGFR	0.77 [0.41–1.45]	0.39 [0.23–0.67]	20/37	12/35	3/37	4/35	31/37	24/35	0/37	10/35
Ning et al. (18)	RCT	NSCLC IIIA	A:Erlotinib(*N* = 53) B:PP-chemo(*N* = 53)	Mut EGFR	N.A.	N.A.	35/53	22/53	6/53	10/53	46/53	43/53	15/53	25/53
Xiong et al. (19)	Non-RCT	NSCLC IIIA	A:Erlotinib(*N* = 15) B:Cisplatin-based chemotherapy (*N* = 16)	Total	0.48 [0.19–1.19]	0.66 [0.22–1.97]	10/15	3/16	0/15	1/16	12/15	8/16	N.A.	N.A.

*NSCLC, non-small cell lung cancer; GC, gemcitabine/carboplatin; PP, pemetrexed/cisplatin; mut, mutatated; OS, overall survival; PFS, progression free survival; OR, objective response; N.A., data not available; NTT, neoadjuvant targeted therapy; NCT, neoadjuvant chemotherapy; AEs, grade 3/4 adverse events*.

### Efficacy

#### Overall Survival

**Figure 2:**
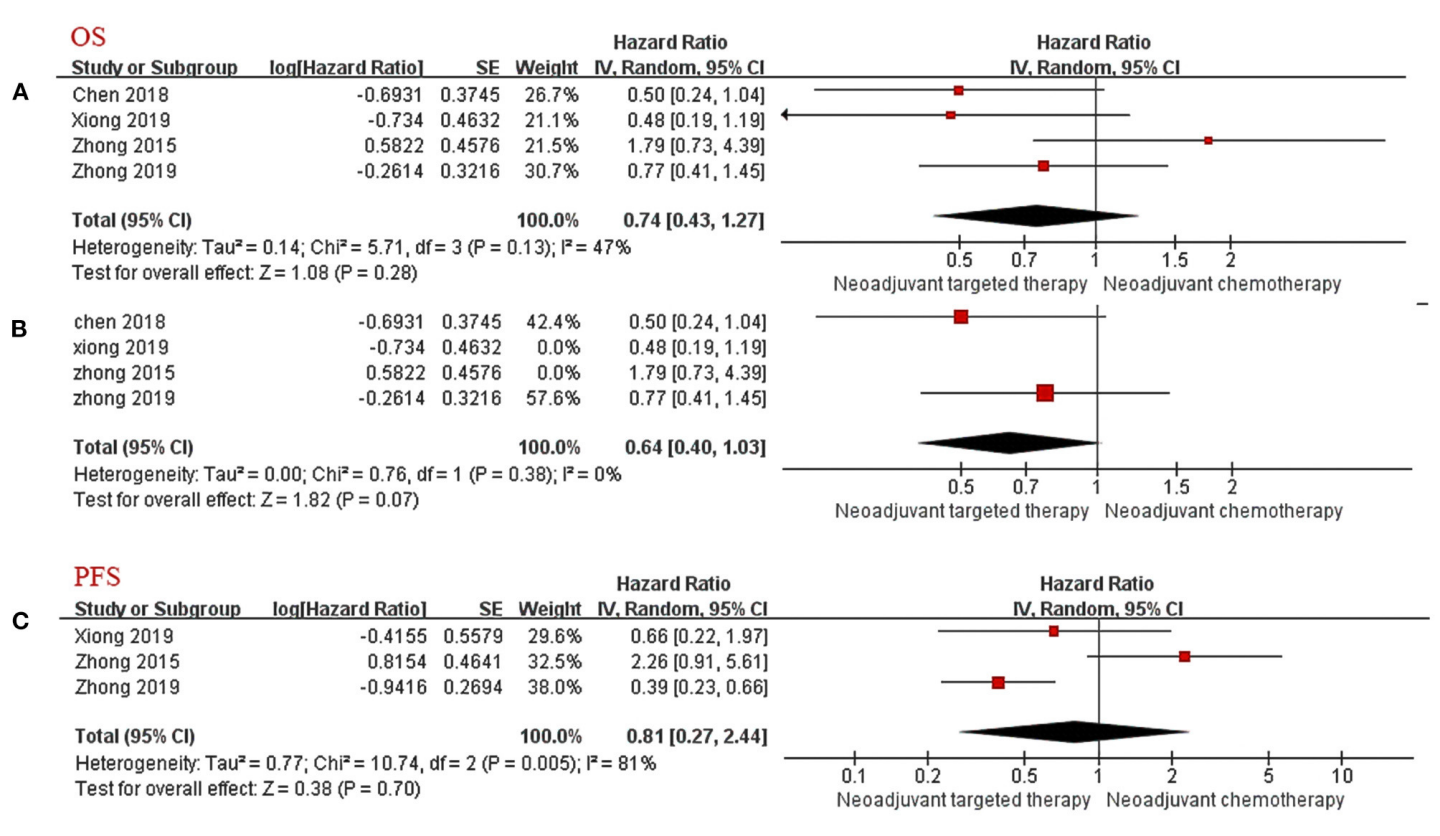
Forest Plot for Overall Survival **(A,B)** and Progression-Free Survival **(C)**. CI, confidence interval; HR, hazard ratio; SE, standard error. ^a^HR < 1 meant a better prognosis in the experimental group (NTT). ^b^*I*^2^ < 25% can choose fixed effect model, otherwise choose random effect model.

Data for the OS were available in four trials (two RCTs and two non-RCT). The results after merging all four studies showed that the difference of survival prognosis between NTT and NCT is not significant (HR 0.74, 95% CI: 0.43–1.27, *I*^2^ = 47%; Figure 2A), and the heterogeneity was large. After excluding non-RCTs, the heterogeneity of the data disappeared (*I*^2^ = 0%). The OS of the NTT arm is slightly higher, but the difference is not significant (HR 0.64, 95% CI: 0.40–1.03, *I*^2^ = 0%; Figure 2B).

#### Progression-Free Survival

Only three articles provided PFS follow-up data. Because of the large heterogeneity, the random effects model was adopted. The results showed no significant difference in PFS between the NTT group and the NCT group (HR 0.81, 95% CI: 0.27–2.44, *I*^2^ = 81%; Figure 2C).

#### Objective Response Rate

**Figure 3:**
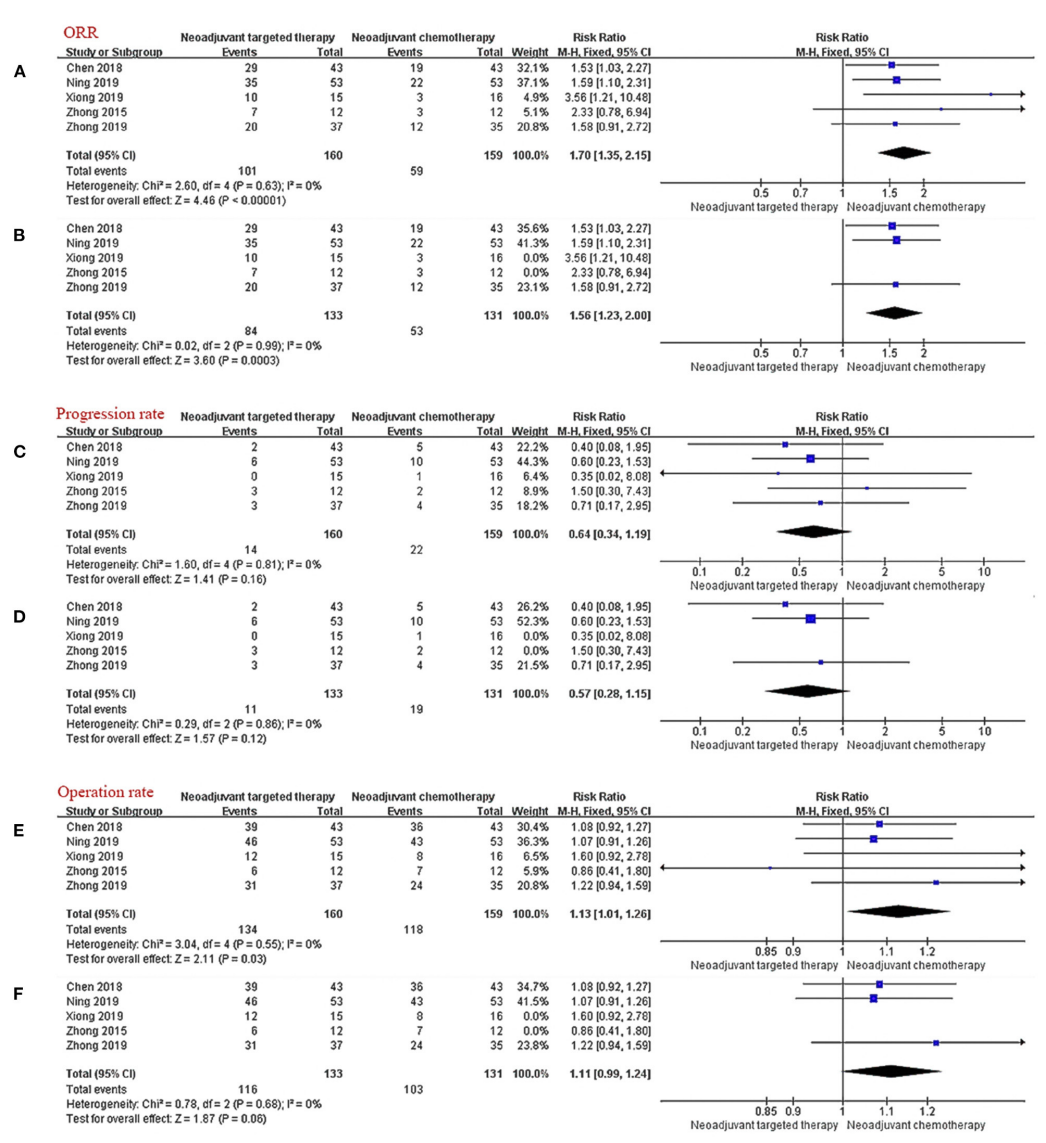
Forest plot for ORR **(A,B)**, progression rate **(C,D)**, and operation rate **(E,F)**. CI, confidence interval; RR, relative risk. ^a^RR > 1 meant that the incident occurred more in the experimental group (NTT). ^b^*I*^2^ < 25% can choose fixed effect model, otherwise choose random effect model.

The consistency of the five studies shows that NTT has a significant advantage in terms of the ORR (RR 1.70, 95% CI: 1.35–2.15, *I*^2^ = 0%; Figure 3A). In the subgroup analysis excluding non-RCTs, the results show that the relative risk for ORR in EGFR mutation-positive stage IIIA NSCLC is 1.56-fold higher with EGFR-TKI than with chemotherapy (RR 1.56, 95% CI: 1.23–2.00, *I*^2^ = 0%; Figure 3B).

#### Progression Rate

All five trials provided follow-up data on progress rates. Very few patients progressed during neoadjuvant therapy, there were ~13.8% in the NCT group and 8.8% in the NTT group. The incidence of progression during treatment in the NTT group was lower than that in the NCT group, but the findings were limited by the small sample size and the difference was not statistically significant (RR 0.64, 95% CI: 0.34–1.99, *I*^2^ = 0%, Figure 3C); subgroup-RR 0.57, 95% CI: 0.28–1.15, *I*^2^ = 0%, (Figure 3D).

#### Operation Rate

All five studies provided surgical data. The operation rate after NTT was ~83.8% and after NCT was 74.2%. NTT was significantly better than NCT in terms of the surgical resection rate for patients with EGFR mutation-positive stage IIIA NSCLC (RR 1.13, 95% CI: 1.01–1.26, *I*^2^ = 0%; Figure 3E). After excluding the data of non-RCT, the advantage of NTT loses statistical significance (RR 1.11, 95% CI: 0.99–1.24, *I*^2^ = 0%; Figure 3F).

### Toxicity

**Figure 4:**
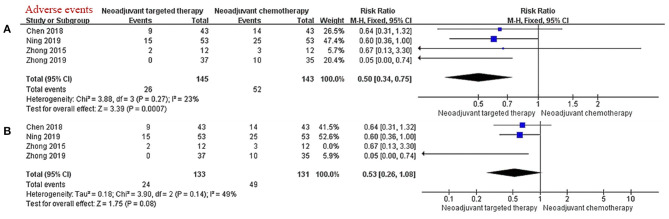
**(A,B)** Forest plot for grade 3/4 adverse events. CI, confidence interval; RR, relative risk. ^a^RR > 1 meant a better ORR in the experimental group (NTT). ^b^*I*^2^ < 25% can choose fixed effect model, otherwise choose random effect model.

There were four studies describing the frequency and nature of AEs during neoadjuvant therapy. The main grade 3/4 adverse event of chemotherapy is myelosuppression, and the main grade 3/4 adverse event of EGFR-TKI is skin rash. The results of the meta-analysis showed that the incidence of grade 3/4 AEs in the NTT group was significantly less than that in the NCT group (RR 0.50, 95% CI: 0.34–0.75, *I*^2^ = 23%; Figure 4A). After excluding data from the non-RCTs and selecting the random effects model, the tolerability advantage of NTT does not reach statistical significance (RR 0.53, 95% CI: 0.26–1.08, *I*^2^ = 49%; Figure 4B).

### Risk of Bias

Fewer than 10 studies included in this meta-analysis, the power of funnel plot asymmetry is too low to distinguish chance from real asymmetry. We used the RevMan version 5.3 to evaluate the quality of eligible study according to the Cochrane Handbook for Systematic Reviews of Interventions, and then the risk of bias for each study is summarized in Table 2.

**Table 2:** Risk of Bias of included studies.

	**Random sequence generation**	**Allocation concealment**	**Blinding of participants and personnel**	**Incomplete outcome data**	**Selective reporting**	**Other bias**
Chen et al. (16)	+	^*^	+	+	+	^*^
Ning et al. (18)	+	^*^	+	+	–	^*^
Zhong et al. (17)	+	^*^	+	+	+	+
Zhong et al. (15)	–	–	+	+	+	^*^
Xiong et al. (19)	–	–	+	+	^*^	^*^

+, Low risk of bias, −, high risk of bias. ^*^, Unclear.

## Discussion

As the efficacy of EGFR-TKI has been confirmed, NCCN Guidelines have now adopted it as a first-line treatment for patients with advanced EGFR mutation-positive NSCLC (5). However, in neoadjuvant setting, there is still controversy about the routine administration of TKI treatment to patients with stage IIIA EGFR mutation-positive NSCLC. This is the first meta-analysis comparing efficacy and safety of NTT and NCT for EGFR mutation-positive stage IIIA NSCLC, and most of the relevant clinical studies are still ongoing.

The results of our meta-analysis suggest that the induction therapy of locally advanced EGFR mutation-positive NSCLC patients can give priority to EGFR-TKI. For example, in our analysis, NTT has the potential to improve the survival prognosis compared with NCT. Although, according to Zhong's literature reports, the survival time of EGFR mutation-negative patient who received NCT was longer than that of EGFR mutation-positive patient who received NTT (15). Although the survival data in the study by Zhong et al. is diametrically opposed to other studies on the forest plot, it is not completely contradictory to our conclusion (15). This advantage may be due to the biological characteristics brought about by EGFR gene mutations, resulting in a worse basic prognosis for patients with mutation-positive (20). Assigning patients to NTT or NCT based on EGFR mutation status may be the most effective strategy.

A meta-analysis of advanced NSCLC suggested that EGRF-TKI treatment can significantly prolong the PFS of EGFR mutation-positive patients (21). In our study, CTONG1103, a large prospective randomized controlled trial, confirmed that TKI can also improve PFS in neoadjuvant setting compared to traditional chemotherapy (17). There is currently no other reliable evidence about PFS.

In each included studies, the absolute value of ORR of each NTT group is higher than that of the NCT. Due to the limitation of sample size, the advantage was not statistically significance in a part of studies. After summarizing the above data, NTT has a consistent advantage over NCT in ORR. The EGFR mutation status can be regarded as a predictive biomarker of tumor regression after EGFR-TKI treatment, and the tumor regression of EGFR mutation-positive patients who received NTT was more significant than that of EGFR mutation-negative patients who received NCT. The NCCN Guidelines recommend that locally advanced patients who have no apparent progress after induction therapy should be followed by surgery and postoperative adjuvant treatment (5). The intense tumor response after TKI treatment echoes the lower progression rate and higher surgery rate during the treatment period in our analysis. Studies have reported that neoadjuvant chemotherapy may cause fusion of tissue planes, tissue edema, or adhesions around the lesion, thereby increasing the difficulty of surgical dissection and tumor resection (16). Unfortunately, the existing literature does not provide sufficient data on R0 resection.

In fact, it is difficult to absolutely compare the survival difference between NTT and NCT in clinical setting. It is possible that patients may have cross-treatment during the adjuvant treatment stage, and this would weaken the survival benefit of NTT and make it difficult to obtain the end point of prognosis improvement. Assuming in a more conservative setting, if there is no significant difference between NTT and NCT for the stage IIIA EGFR mutation-positive patient's survival prognosis, choosing a treatment with more tolerable adverse effects is also a quality improvement direction worth considering. Our research found that the EGFR mutation-positive stage IIIA NSCLC patients are extremely sensitive to the NTT strategy, meanwhile, the incidence of grade 3/4 AEs in the NTT group was significantly lower than that of NCT. Consistent with previous reported results, the main adverse event of NTT is skin rash, and the main adverse events of NCT are myelosuppression and gastrointestinal reaction (22). In clinical practice, the toxicity associated with myelosuppression may be ignored by patients because it has no direct symptoms in the early stage (23). The gastrointestinal reaction often affects the quality of life and causes the patient to visit the doctor repeatedly (24). However, myelosuppression may be associated with higher risk complications. Although the analysis results show acceptable toxicity of TKI, the conclusion is based on idealized test conditions. For example, patients included in the trial need to have no significant liver or kidney damage. In actual clinical work, most patients with lung cancer are elderly, often with various underlying diseases, which reduces their tolerance to drug toxicity.

In the United States where the EGFR mutation rate is low, the effect of TKI as an induction therapy may be limited. Following the NCCN guidelines for preoperative induction chemotherapy may be a better choice. However, for East Asian where the EGFR-mutation rate is as high as 50%, we recommend tumor or mediastinal nodal biopsy and EGFR-mutation assessment before surgery to improve patient prognosis through personalized targeted therapy.

## Limitations

This report concerns a meta-analysis, and it has some limitations. First of all, we do not have detailed data for each patient. Second, some data were not directly provided in the articles, and we could only estimate the missing data by means of a chart. Third, this direction in therapy is still an emerging topic. At present, many similar clinical trials have not yet completed follow-up and the data that can be obtained are limited. Fourth, although the EGFR-TKI used in all test groups was erlotinib, the chemotherapy regimen of the control group varied slightly between different trials. Fifth, in actual clinical settings, postoperative patients receiving adjuvant treatments cannot be compulsorily controlled, and there may be partial cross-treatments. The prognostic evaluation bias caused by this situation cannot be estimated. The views provided in this article are only for reference, and the establishment of the best treatment model requires more large-scale clinical trials.

## Conclusions

For the comparison of NTT and NCT treatment strategies in patients with EGFR mutations in stage IIIA, NTT has a trend to improve OS; no difference in PFS was found; NTT has a higher ORR; the incidence of grade 3/4 AEs in the NTT group is lower. In neoadjuvant setting, targeted therapy has a definitive effect on patients with EGFR mutation-positive stage IIIA NSCLC and is even better than chemotherapy in terms of toxicity and tumor response rate.

## Data Availability Statement

The original contributions presented in the study are included in the article/Supplementary Material, further inquiries can be directed to the corresponding authors.

## Author Contributions

BC and JS designed the direction and ideas of the research. Data collection and analysis were performed by DC, ZJ, and JZ. The first draft of the manuscript was written by DC and all authors commented on previous versions of the manuscript. CX, KZ, and YR were responsible for the proofreading. All authors read and approved the final manuscript.

## Conflict of Interest

The authors declare that the research was conducted in the absence of any commercial or financial relationships that could be construed as a potential conflict of interest.

## Publisher's Note

All claims expressed in this article are solely those of the authors and do not necessarily represent those of their affiliated organizations, or those of the publisher, the editors and the reviewers. Any product that may be evaluated in this article, or claim that may be made by its manufacturer, is not guaranteed or endorsed by the publisher.

## Abbreviations

EGFR-TKI: epidermal growth factor receptor-tyrosine kinase inhibitor
NCT: neoadjuvant chemotherapy
NSCLC: non-small cell lung cancer
NTT: neoadjuvant targeted therapy
ORR: overall objective response rate
OS: overall survival
PFS: progression-free survival
AEs: adverse events.
