# Supplementary material for: Efficacy and Safety of Neoadjuvant Targeted Therapy vs. Neoadjuvant Chemotherapy for Stage IIIA EGFR-Mutant Non-small Cell Lung Cancer: A Systematic Review and Meta-Analysis
Source: Front Surg. 2021 Aug 19;8:715318. doi: 10.3389/fsurg.2021.715318 (PMC8417411; doi:10.3389/fsurg.2021.715318)
Supplement: Supplementary file 1 [file Data_Sheet_1.docx]

Search strategies for pubmed：

(((((("Lung Neoplasms"[Mesh]) OR "Carcinoma, Non-Small-Cell Lung"[Mesh]) OR (NSCLC[Title/Abstract])) OR (((((((((((((Neoplasia[Title/Abstract]) OR (Neoplasias[Title/Abstract])) OR (Neoplasm[Title/Abstract])) OR (Neoplasms[Title/Abstract])) OR (Tumors[Title/Abstract])) OR (Tumor[Title/Abstract])) OR (Cancer[Title/Abstract])) OR (Cancers[Title/Abstract])) OR (Malignancy[Title/Abstract])) OR (Malignancies[Title/Abstract])) OR (Carcinoma[Title/Abstract])) OR (Carcinomas[Title/Abstract])) AND ((Lung[Title/Abstract]) OR (Pulmonary[Title/Abstract])))) AND (("Neoadjuvant Therapy"[Mesh]) OR (((((((neoadjuvant[Title/Abstract]) OR (neo adjuvant[Title/Abstract])) OR (preoperative[Title/Abstract])) OR (pre operative[Title/Abstract])) OR (pre surgical[Title/Abstract])) OR (presurgical[Title/Abstract])) OR (Induction[Title/Abstract])))) AND ((("Molecular Targeted Therapy"[Mesh]) OR ("Genes, erbB-1"[Mesh])) OR (((((((((((((((((Targeted[Title/Abstract]) OR (EGFR[Title/Abstract])) OR (Epidermal Growth Factor Receptor[Title/Abstract])) OR (almonertinib[Title/Abstract])) OR (Osimertinib[Title/Abstract])) OR (Gefitinib[Title/Abstract])) OR (afatinib[Title/Abstract])) OR (Erlotinib[Title/Abstract])) OR (icotinib[Title/Abstract])) OR (dacomitinib[Title/Abstract])) OR (TKI[Title/Abstract])) OR (tyrosine kinase inhibitor[Title/Abstract])) OR (tyrosine kinase inhibitors[Title/Abstract])) OR (TKIs[Title/Abstract])) OR (erbB1[Title/Abstract])) OR (erbB 1[Title/Abstract])) OR (v erbB[Title/Abstract])))) NOT (((((((((("case reports"[Publication Type]) OR ("comment"[Publication Type])) OR ("letter"[Publication Type])) OR ("editorial"[Publication Type])) OR ("news"[Publication Type])) OR (comment[Title])) OR (case report*[Title])) OR (letter[Title])) OR (editorial[Title])) OR (news[Title]))
